# Late Complications in acute Leukemia patients following HSCT: A single center experience

**Published:** 2016-01-01

**Authors:** Mohammad Vaezi, Cyrous Gharib, Maryam Souri, Ardeshir Ghavamzadeh

**Affiliations:** 1 Hematologist- Oncologist, Hematology- Oncology and Stem Cell Transplantation Research Center, Tehran University of Medical Sciences, Tehran, Iran; 2 Hematologist- Oncologist, Gilan University of Medical Sciences, Gilan, Iran; 3 Hematology- Oncology and Stem Cell Transplantation Research Center, Tehran University of Medical Sciences, Tehran, Iran; 4 Professor of Medicine, Hematology-Oncology and Stem Cell Transplantation Research Center, Tehran University of Medical Sciences, Tehran, Iran

**Keywords:** Hematopoietic stem cell transplantation (HSCT), Late complications, TBI / BuCy regimens

## Abstract

**Background:** Hematopoietic stem cell transplantation (HSCT) is currently the only curative treatment for acute leukemia. As HSCT improves the long-term survival, it is necessary to assess the late-onset complications affecting the quality of life following HSCT.

**Subjects and Methods:** The study included 122 patients (65 male, 57 female) with leukemia (72 AML and 50 ALL) who received transplants from fully- matched siblings, unrelated donors and unrelated cord blood donors between February 2013 and August 2014 in Shariati Hospital. All study participants were over 18 years of age and had the minimum and maximum survival of 2 and 5 years, respectively. Patients who received HLA-haploidentical SCT were excluded from the study. All allogeneic recipients received busulfan and cyclophosphamide as conditioning regimen. Nobody received TBI-based conditioning regimen in this study. Patients were evaluated for cardiovascular, vision, psychological, endocrine, fertility problems and secondary malignancies one year after transplantation.

**Results** **:** Data were analyzed using SPSS 15.0. Mitral and tricuspid regurgitation (TR/MR) were the most common cardiac complications (n=12, 10.5%).Thirty-nine percent of patients had psychological problems, especially depression (34%). Cataract was observed in 13% of patients and 34% complained of dry eye. Symptomatic pulmonary changes were found in 13 patients (10.6%). None of the HSCT survivors had experienced fertility before study entry. According to LH and FSH levels, 15% and 9% of females had ovarian failure, respectively. Testosterone level was less than normal in 49(84%) men and, according to their FSH and LH level, 20 (41%) had secondary hypogonadism and 29 (59%) had primary gonadal dysfunction.

**Conclusion:** The results showed that patients who received Bu/Cy conditioning regimen experienced fewer late side effects such as cataract formation and hypothyroidism, compared to previous studies using TBI-based conditioning regimen.

## Introduction

 Leukemia is a rapidly progressive and lethal disorder. Hematopoietic stem cell transplantation (HSCT) is one of the most effective therapeutic modalities for the treatment of AML and ALL patients.^1^ In this method, leukemia patients who achieve cytogenetic remission by chemotherapy are considered candidates for HSCT. Conditioning regimens are used to prepare recipients for HSCT. The first conditioning regimens used for preparation of patients contained only total body irradiation,^2^ but then, chemotherapy was administered alone or in combination with TBI.

High dose TBI -based conditioning regimen causes abnormalities of growth and development, pulmonary complications, gonadal dysfunction (Hypothyroidism) and secondary malignancies in pediatric patients. Chemotherapy-based conditioning regimens without TBI are currently used to treat patients with leukemia in transplant centers worldwide. The most commonly used non-TBI alternative is busulfan and  cyclophosphamide (Bu/Cy).^3^^,^^4^

The results of several studies have shown that the Bu/Cy regimen was better tolerated in patients with leukemia, compared with those who were treated with TBI and cyclophosphamide.^3^^,^^4^ Meanwhile, there was no significant difference in the risk of GvHD and outcome of transplantation between recipients of Bu/Cy and CyTBI (cyclophosphamide/TBI).^5^

According to previous studies, late complications in HSCT recipients are correlated with type of disorder, pre-transplant conditioning regimens, especially TBI, age, sex and underlying disease.

Pulmonary complications are commonly observed after HSCT. The results of a study conducted in Turkey (Ankara) showed decreased DLCO, FBV and FEV1 in 110 pediatric patients who underwent HSCT between 1996 and 2006.^6^ Another study in Switzerland on "Examining Late Effects following HSCT" showed that late complications are associated with transplant conditioning regimen, infectious complications after HSCT, chronic GvHD and its treatment.^7^ Pulmonary complications, obstructive and restrictive lung diseases usually occur between 3 to 24 months or even more years after HSCT. It has been mainly associated with TBI, GvHD and age of patients at the time of transplantation.^8^^,^^9^

Other studies in European countries revealed the impact of late side effects such as chronic GvHD, psychiatric, cardiac, ophthalmic and pulmonary complications as well as gonadal dysfunction (infertility and thyroid) on the quality of life in HSCT survivors. The survey results were cited in a review article entitled “non-Malignant Late Effects after HSCT”.^10^ The development of cataract in HSCT recipients is closely correlated with TBI and steroid treatment. High-dose TBI (≥10 Gy) is associated with a higher risk of cataract (up to 80%). The risk of cataract development is 20% in patients receiving conditioning regimens of Bu/Cy or cyclophosphamide alone.^11^

Hypothyroidism is one of the most frequent late complications after HSCT. The risk of developing hypothyroidism in patients receiving Bu/Cy is about 12%. The average time to develop hypothyroidism is 4 years after HSCT.^12^

Survivors of HSCT are at a higher risk for developing cardiovascular complications. Prevention and treatment of cardiovascular complications in the transplant patients are the same as the non-transplant patients. Studies have reported that 22% of HSCT survivors develop coronary artery abnormalities 25 years after transplantation.^13^^,^^14^

The incidence of solid tumors and late adverse effects of HSCT is higher in transplant recipients. There are multiple factors that may predispose to the development of secondary solid tumors after HSCT including TBI-based conditioning regimens, initial disease and male sex.^15^ There is an increase in the risk of secondary solid tumors following immunosuppressive treatment and incidence of GvHD.^17^ These secondary malignancies can be categorized as post-transplant lymphoproliferative disorders (PTLD), hematologic malignancies and solid tumors.^16^

## SUBJECTS AND METHODS

This study included 122 patients with leukemia (AML and ALL) who were transplanted using the busulfan- cyclophosphamide (Bu/Cy) preparative regimen in Shariati Hospital between February 2013 and August 2014. Transplant patients had the minimum and maximum survival of 2 and 5 years, respectively. Patients who were under 18 years old or received HLA- haploidentical SCT were excluded from the study (Ethical committee No: ir.tums.horcsct.1394.103.13).

Prior to HSCT, all patients were routinely examined by cardiologist, psychiatrist, ENT specialist, dentist, forensic medical specialist, pulmonologist and if necessary, by other consulting physicians. Complementary tests such as spirometry, echocardiography, CT scan of lung, brain, paranasal sinuses, abdomen and pelvis as well as laboratory tests including organ function and viral studies of donor and recipient were also performed. Clinical and laboratory data of patients were then recorded. At our center, all acute leukemia patients received myeloablative conditioning regimen of BU/ CY and none of the patients received TBI-based conditioning regimen.

One year after transplantation, patients were followed-up for ophthalmic, cardiovascular, pulmonary, endocrine, fertility and psychological complications as well as second malignancies. All acute leukemia patients who survived longer than 1 year after transplant were examined by above- mentioned specialists and performed related laboratory tests including spirometry, echocardiography, fundoscopic slit lamp examination*,* TSH, T4, T3RU, FSH, LH, testosterone, ECG and CT scan of lung. Those patients who completed requested examinations and laboratory tests were enrolled in the study.

Pulmonary complications including airway and lung parenchyma are more commonly observed after HSCT. The most common late complications include bronchiolitis obliterans (BO) and bronchiolitis obliterans organizing pneumonia (BOOP).^17^ BO was clinically diagnosed with the following criteria:

(1) FEV_1_/FVC ratio < 0.7 and FEV_1_< 75% of predicted value; (2) evidence of air trapping or small airway thickening or bronchiectasis in HRCT; and (3) absence of infection in the respiratory tract.^18^

Late cardiac complications including congestive heart failure, cardiomyopathy, valvular disease or arrhythmia were detected by ECG and echocardiography.

The two most common late complications affecting the anterior segment are cataract formation and kerato-conjunctivitis sicca syndrome which were diagnosed by slit lamp and fundoscopic examination by ophthalmologist. Endocrine and fertility abnormalities were defined as TSH>5 u/ml, abnormal FSH, LH and testosterone results.

Psychiatric disorders were assessed according to DSM-IV criteria by psychiatrist.

New post-HSCT malignancies that are related to primary therapy for cancer are rare but devastating complications. All results were recorded in checklists. Data were analyzed using the SPSS software version 15.0* (*SPSS*, *Inc*.; *Chicago*, *IL*, *USA*).*

## Results

 A total of 122 patients with acute leukemia were enrolled in the study, 72 (59%) had AML and 50 (41%) had ALL. There were 65 (53%) males and 57 (47%) females. In this series, 97.5% of patients received allogeneic transplantation, while 2.5% underwent autologous transplantation. DLI was not given to 96% of patients, while 3% received DLI once and 1% twice during treatment. Stem cells were derived from bone marrow (1%) and peripheral blood (99%) in this study.

83.5% of patients were transplanted in first complete remission (CR1), 14% in CR2 and 1% in CR3. 1.5% of transplantations were also performed in patients with primary induction failure.

Patients underwent transplantation from HLA-identical siblings (89.5%), other related (6.5%) and unrelated donors (1.5%). As mentioned above 2.5% received autologous transplantation. 95% of patients had HLA-matched donor and 2.5% had one-locus mismatched donor.

**Late Complications**

**Ophthalmic problems**: Sicca syndrome (dry eye) was the most frequent late ophthalmic complication after HSCT, which occurred in 34% ) n=41) of patients in this study. 15 (12.5%) patients developed cataract.

**Endocrine**: 8% (n=10) of patients developed primary hypothyroidism. None of the HSCT survivors had already experienced fertility and gonadal dysfunction. 17 of 57 female patients did not take LH and FSH tests and 4 patients were over 50 years. According to LH and FSH levels, 15% and 9% of patients had ovarian failure, respectively. 7 of 65 male transplant patients did not do hormonal tests. Testosterone level was less than normal in 49 (84%) men and according to their FSH and LH levels, 20 (41%) had secondary hypogonadism and 29 (59%) had primary gonadal dysfunction.

**Pulmonary complications**: Obstructive and restrictive pulmonary diseases were detected in 15% and 6% of HSCT recipients, respectively. BOOP was identified in 6 (5%) patients.

**Cardiac**: Mitral and tricuspid regurgitation (MR/TR) were the most common cardiac complications among patients (n=12, 10.5%). Pericardial effusion occurred in 1 (1%) patient and right bundle branch block (RBBB) was observed in 1 (1%) patient.

**Psychological disorders:** Transplant-related psychological distress is very common among recipients. The present study has documented psychological disorders in 39% of HSCT survivors, of whom 87% were diagnosed with depression and 13% with anxiety disorders and insomnia. One patient presented with convulsion.

**Secondary solid tumors**: One patient was affected by astrocytoma. Also, 37 (30%) patients experienced chronic and non-pulmonary GvHD, including skin and liver GvHD.

## Discussion

 The 2-year cumulative incidence of late pulmonary complications was 10% among 438 patients surviving more than 3 months in the retrospective study conducted by Patriarca et al.^19^ Hartsell et al.^20^ retrospectively compared pulmonary complications in patients conditioned with cyclophosphamide and TBI (CY/TBI) and busulfan and cyclophosphamide (BU/CY). Late pulmonary events (occurring >45 days after transplant) were significantly higher in the CY/TBI, compared to BU/CY (n=15 versus 4; P=.04). In a meta-analysis done by Gupta et al. CY/TBI was associated with a moderate though non-significant increase in the risk of clinically significant pulmonary complications, compared to BU/CY.^21^ In our study, 21% of patients developed spirometric abnormalities. BOOP was identified in 6 (5%) patients. Pulmonary symptoms were found in 13 (10.6%) patients. It may be due to busulfan toxicity on lung or subsequent GvHD.

The probability of developing cataract after fractionated TBI is about 30% at 3 years. This incidence may be more than 80% 6 to 10 years after HSCT.^22^^,^^23^ Several studies have shown cataract formation in more than 80% in the single-dose TBI group and <20% in the no TBI group.^11^ In our study, cataract formation was only found in 12.5% of patients. This finding may be due to short period of follow-up, but it still seems to be much less than TBI-based conditioning post-HSCT cataract formation. In prospective studies of the incidence of cataracts, patients who treated with Cy/TBI had a higher incidence of cataract formation than those received Bu/Cy.^24^

In one study, 85% of the patients receiving TBI developed azoospermia versus 51% in the no TBI group.^25^ The majority of HSCT survivors become infertile although HSCT without total body irradiation can spare fertility in nearly one-third of men and women. Semen analysis was not done in our study and we just assessed fertility in our patients following HSCT. No pregnancy happened in HSCT recipients.

Studies show that 7% to 15.5% of patients will demonstrate subclinical hypothyroidism during the first year post HSCT.^24^ 90% of patients who have received single-dose TBI need levothyroxine as hormone replacement therapy, while 14–15% of patients treated by fractionated TBI and a small number of patients conditioned with Bu/Cy need that remedy.^24^ In our study, 8% of patients developed primary hypothyroidism. Like other studies, our findings favor the use of non-TBI-based conditioning regimen.

The median time from HSCT to presentation of solid tumors is between 5 to 6 years. They account for 5% to 10% of deaths among HSCT recipients who survive two years or longer.^16^ The cumulative incidence of invasive solid tumors is about 8% at 20 years.^24^ We found just one patient with solid tumor in our study that may be due to short follow-up period and small number of patients evaluated.

## CONCLUSION

 In order to improve the quality of life and overall survival in HSCT recipients, careful assessment of treatment-related complications should be part of regular follow-up of HSCT survivors. Diagnostic and therapeutic interventions must also be taken into account to prevent, early diagnosis and treatment of late effects of  HSCT. Survivors should be screened for evidence of hypothyroidism at the periodic health examination. There also should be regular periodic examination of the ocular, cardiovascular, pulmonary and mental status. Due to high risk of infertility in survivors of HSCT, it is recommended to store male’s sperm and female’s ovule prior to HSCT to preserve fertility in adult patients with leukemia. In addition, our results imply that non-TBI based conditioning regimen has fewer late complications at least in acute leukemia patients who have undergone HSCT. These results should be confirmed by studies involving larger sample sizes.
